# Cost-utility of respiratory syncytial virus prefusion F (RSVpreF) maternal vaccine for disease prevention among colombian infants

**DOI:** 10.1186/s12913-026-15139-0

**Published:** 2026-08-03

**Authors:** Jaime Ordóñez, Beatriz Ospina

**Affiliations:** 1 True Consulting S.A.S., Medellín, Colombia; 2 Independent Pediatrician and Researcher, Rionegro, Colombia

**Keywords:** Respiratory syncytial virus infections, Vaccination, Cost-benefit analysis, Pregnancy, Health economics, Infant, Newborn

## Abstract

**Background:**

Respiratory Syncytial Virus (RSV) is one of the leading causes of acute lower respiratory tract infections in Colombia, therefore, as no widespread antiviral exists, maternal immunization using RSV prefusion F protein vaccine (RSVpreF) represents a promising strategy. This study aims to evaluate the cost-utility of the RSVpreF maternal vaccine in preventing RSV infections among Colombian infants.

**Methods:**

We used a Markov model to simulate a Colombian birth cohort over a lifetime horizon, comparing maternal RSVpreF vaccination with no intervention, reflecting the current standard of care in Colombia. The model incorporated RSV-related outpatient visits, hospitalizations, and deaths. We collected data from clinical trials, peer-reviewed literature, and Colombian national health databases. Costs were analyzed from the perspective of the Colombian healthcare system, adjusted to 2024 USD, and we applied a 5% discount rate to both costs and outcomes. We used a willingness-to-pay (WTP) threshold of $ 7,491 per quality-adjusted life year (QALY). Sensitivity analyses, including one-way and probabilistic sensitivity analyses, were performed to evaluate the robustness of the results.

**Results:**

Maternal RSVpreF vaccination was associated with a reduction of approximately 25,781 RSV-related cases and an increase of 2,315 QALYs. The incremental cost-effectiveness ratio (ICER) was $ 2,322 per QALY, well below the WTP threshold. Sensitivity analyses identified vaccine effectiveness, vaccine cost and RSV hospitalization rates as the primary drivers of cost-effectiveness. The probabilistic sensitivity analysis indicated an 83.5% probability that the intervention would be cost-effective at the established WTP threshold.

**Conclusion:**

Maternal RSVpreF vaccination is a cost-effective intervention in Colombia, resulting in significant reductions in RSV-related morbidity and mortality, as well as in the economic burden on the healthcare system. The findings support the inclusion of maternal RSV vaccination in national immunization programs, promoting health equity in underserved communities.

**Clinical trial number:**

Not applicable.

**Supplementary Information:**

The online version contains supplementary material available at https://doi.org/10.1186/s12913-026-15139-0.

## Introduction

Respiratory syncytial virus (RSV) is one of the leading causes of morbidity and mortality in neonates and infants worldwide. In Colombia, this respiratory infection poses a significant threat to public health due to its high transmissibility and the high costs of treatment, especially in severe cases requiring hospitalization or intensive care [1, 2]. RSV infection is the most frequent cause of bronchiolitis and pneumonia in children under one year of age, being particularly dangerous in premature neonates and infants with pre-existing respiratory or cardiovascular comorbidities [3].

Globally, RSV infections generate a high clinical and economic burden. It is estimated that approximately 33 million episodes of RSV lower respiratory infection occur each year in children under five years of age, resulting in more than 3.6 million hospitalizations and 100,000 deaths, mainly in middle- and low-income countries [4]. In Colombia, the high rate of infections in the infant population is associated with a significant economic impact on the health system, driven by the costs associated with prolonged hospitalizations, treatment in intensive care units, and the use of respiratory support therapies, such as oxygen therapy and mechanical ventilation [2].

Treatment of severe RSV infections is costly, placing significant strain on health system resources. In addition, there are no widely effective antivirals, so prevention through vaccination becomes a fundamental strategy to mitigate the disease’s clinical and financial impact [5]. In this context, maternal vaccination against RSV offers an innovative preventive approach, transferring antibodies to the fetus during gestation and providing passive protection to the newborn during the first months of life, when it is most vulnerable to severe infections [6].

The introduction of this vaccine aligns with Colombia’s public health policy, which seeks to improve child health indicators by reducing hospitalizations and neonatal mortality from preventable causes. Implementing an RSV vaccination program for pregnant women could reduce the incidence of respiratory infections in neonates and infants, thereby alleviating the economic burden on the health system. Previous studies show that vaccination reduces clinical outcomes and optimizes budgetary resources by avoiding the high costs of hospital management and associated complications [7–9]. Other emerging preventive strategies, such as long-acting monoclonal antibodies administered to infants, further expand the RSV prevention landscape, although their delivery platforms and target populations differ from those of maternal immunization.

This study aims to perform a cost-utility analysis of the RSVpreF maternal vaccine in Colombia, evaluating its potential to prevent RSV-associated lower respiratory tract illness (LRTI) in neonates and infants. The information obtained will provide a solid scientific basis for public health decision-making, thereby justifying the implementation and financing of vaccination programs for this population.

## Methods

We developed a population-based Markov model to evaluate the clinical and economic impact of maternal RSV prefusion F (RSVpreF) vaccination in Colombia against no vaccination. No other licensed maternal RSV vaccine was available as an active comparator. Infant monoclonal antibodies, such as nirsevimab, were not included because it represent a distinct preventive strategy with a different target population and delivery platform (postnatal vs. antenatal) and were therefore not considered direct comparators [10]. Moreover, an indirect comparison would require formal evidence synthesis across trials with differing designs, enrolled populations, and follow-up windows, which was beyond the scope of this policy-focused evaluation and is consistent with national immunization decision-making processes that evaluate maternal vaccination programs against the current standard of care. The model was developed from the healthcare system perspective.

The model simulated a cohort of newborns and the RSV-related outcomes from birth up to 1 year of life. The model follows the cohort throughout their entire lifetime. Following Colombian health economic evaluation standards, a 5% discount rate was applied to both costs and health outcomes [11]. The model included health states representing outpatient RSV-related events, inpatient hospitalizations, and RSV-related deaths (Fig. 1).

**Fig. 1:**
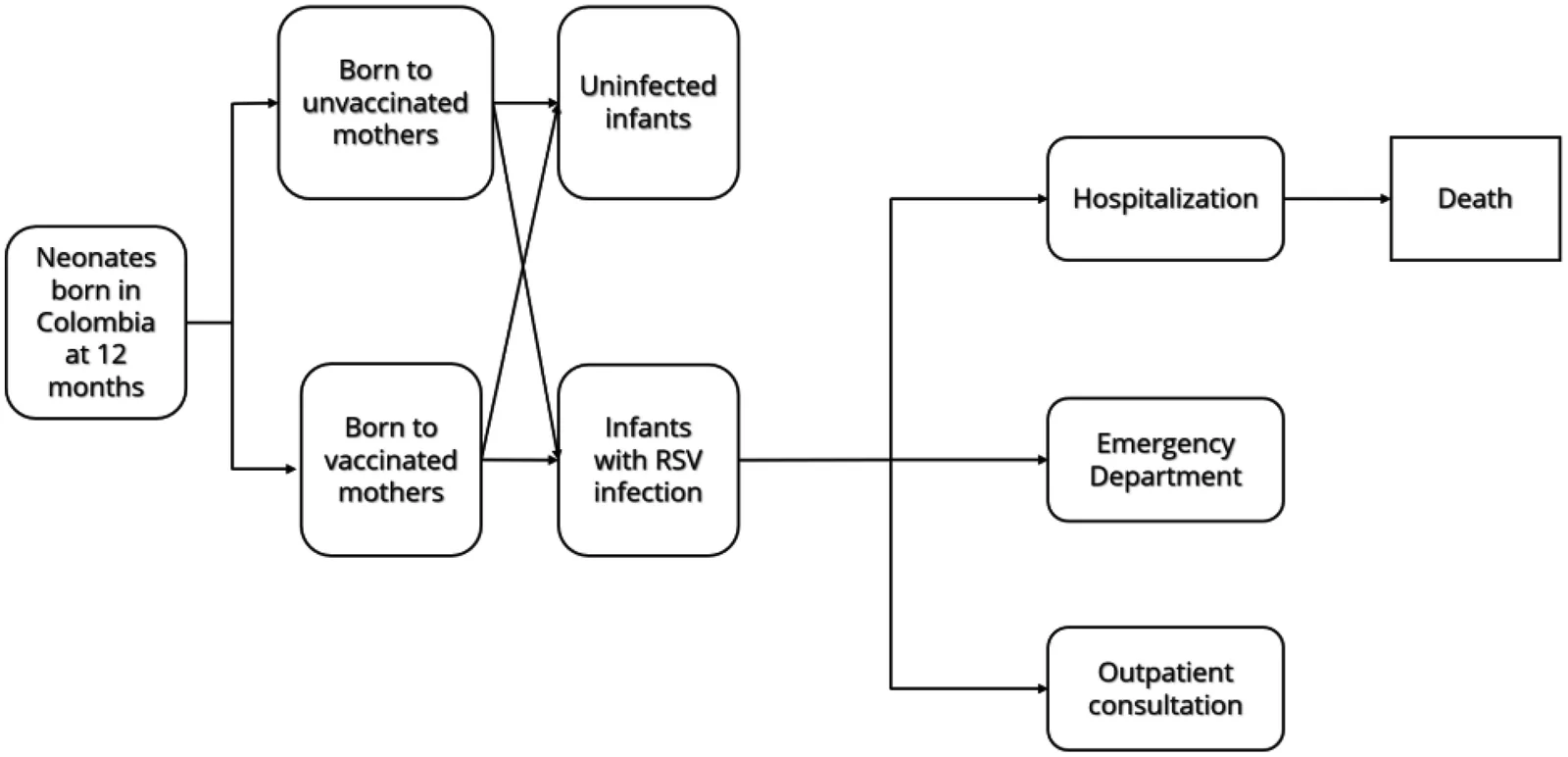
Model structure. schematic of the included health states and the economic model structure

Model inputs integrate data from multiple sources. Epidemiological data were primarily derived from international literature focused on developing and Latin American countries, due to the limited availability of national estimates. Healthcare costs for inpatient and outpatient RSV data were obtained from local studies that ensure appropriate representation of the Colombian context. Vaccine effectiveness and utility were incorporated based on randomized clinical trials and external studies, as no local data were available for these parameters. In the following sections, a detailed description of each input is presented.

## Model input parameters

### Incidences

We derived hospitalization RSV incidence and age-specific rates from estimates presented by Wang et al. [12] for developing countries. Incidence rates for emergency department (ED) visits and outpatient consultations were obtained by averaging the estimates from the systematic review by Heemskerk et al. [13]. Notably, infants under six months exhibit higher susceptibility, with incidences significantly higher than those of infants six months or older. Table 1 presents the annual RSV rates per 1,000 children, stratified by age at the time of consultation and site of patient care.

**Table 1:** RSV incidences: RSV rates by 100.000 inhabitants, adjusted by age in months at the time of the visit and type of visit

Age	Hospital	ED visit	Outpatient visit
< 1 month	51,1	111,5	190,0
1 - < 2 months	51,1	111,5	190,0
2 - < 3 months	51,1	111,5	190,0
3 - < 4 months	51,1	111,5	190,0
4 - < 5 months	51,1	111,5	190,0
5 - < 6 months	51,1	68,0	173,8
6 - <7 months	18,9	68,0	173,8
7 - < 8 months	18,9	68,0	173,8
8 - < 9 months	18,9	68,0	173,8
9 - < 10 months	18,9	68,0	173,8
10 - < 11 months	18,9	68,0	173,8
11 - < 12 months	18,9	68,0	173,8

The model assumes that the 100% of the above RSV incidence in hospitalized setting for all newborns is associated with LRTI; for ED visits and outpatient consultation this proportion is obtained from Rainisch et al. [14], with values of 65% and 50% for infants among 0–5 months and 6–11 respectively in ED visits, and 65% and 30% for the same age groups in outpatient. To adjust for gestational age, we applied relative risks for RSV development based on age in months at the visit, following Rha et al. [15]. Table S1 details these relative risks, showing that preterm infants have a significantly higher susceptibility to severe RSV compared to full-term infants.

### Population

The population of this study includes all neonates in Colombia in 2025, which, based on estimates of births in the country since 2017 (when births began to decrease sharply year after year), would be 429,363. Likewise, the model includes the estimate of fetal deaths in the country for 2025 to adjust costs accordingly, since these mothers would also receive the vaccine. The forecast of fetal deaths in Colombia for 2025 would be 19,140, which means that the population of pregnant women in 2025, based on estimates from vital statistics in the country [16], would be approximately 425,327; based on this information, it was identified that approximately 99.1% of pregnant women would deliver 100% of births. The model also includes the relative frequency distribution of gestational age in weeks (wGA) for neonates.

### Mortality rates

Mortality data for infants < 1 year in the general population were sourced from DANE’s microdata on vital statistics, categorized by age at death: 7.38 per 1,000 for infants under one month, 2.97 for those aged one to six months, and 1.31 for those aged six to twelve months. Lacking specific local data, the RSV-specific case fatality ratio, estimated at 1.7 per 100 cases of RSV, was based on published data for Argentina [17]. Table S2 presents the age-specific relative risk of RSV-related mortality.

### Vaccine effectiveness

We used vaccine efficacy data from the MATISSE clinical trial, stratified by disease severity and infant age [9]. The model assumes RSVpreF is ineffective if infants were born within the two weeks post-vaccination, as antibody titers would not reach protective levels, or among infants born before 32 weeks of gestation. We assumed initial vaccine effectiveness at birth at 84.4% for preventing severe RSV cases requiring hospitalization and 70.5% for milder cases managed in outpatient or emergency settings. This effectiveness was also applied to late preterm infants. Figure 2 outlines the efficacy and waning assumption by month of age and care settings. Vaccine efficacy waning is extracted from the trial and extended linearly to 12 months according to evidence on the kinetics and decay of maternal antibodies transferred to the fetus after vaccination [18–20]. However, in the scenario analysis, it is desired to set it to 6 months based on the MATISSE study.

**Fig. 2:**
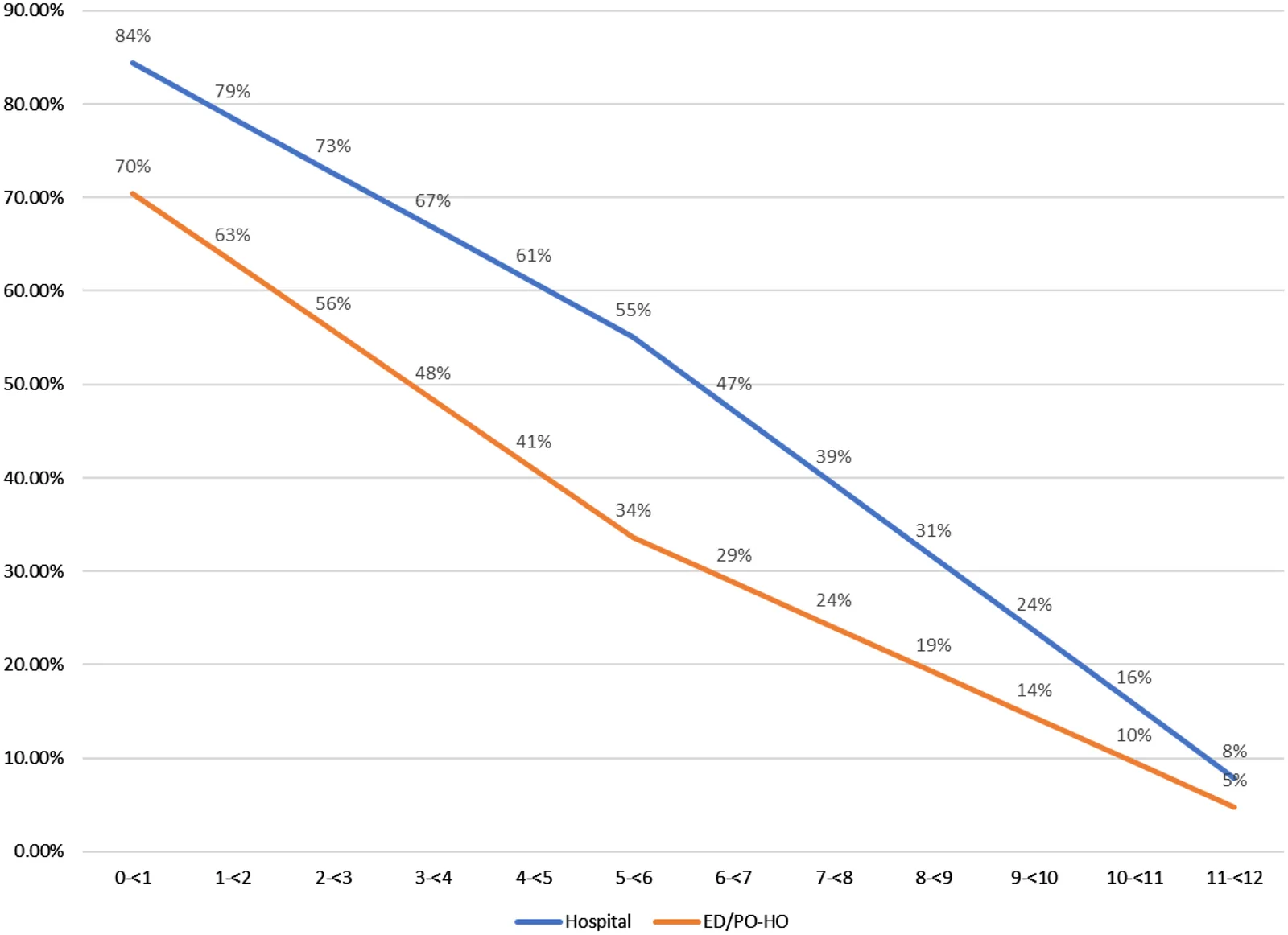
Vaccine effectiveness. effectiveness rate against RSV according to age in months and care setting

### Maternal vaccination coverage

The model assumes that 86.7% of pregnant women will be vaccinated, based on influenza vaccination coverage among pregnant women in 2023 [21]. Given Colombia’s tropical climate with no well-defined seasons, RSV circulates year-round, so the vaccination strategy is assumed to be year-round, with no seasonal focus. The model assumes that no women will be vaccinated before 24 weeks of gestational age (wGA), that 90% will receive a vaccine between 24 and 32 weeks inclusive (10% in each wGA), and the remaining 10% will be vaccinated between 33 and 36 weeks inclusive (2.5% in each wGA).

### Cost inputs

Costs were valued in 2024 USD, using Colombian healthcare tariffs, Pan American Health Organization (PAHO) pricing data, and an exchange rate of 4,020 COP = 1 USD. The RSVpreF vaccine cost was $49 [22], plus a $1 administration fee. Table 2 provides a detailed breakdown of healthcare costs associated with RSV infection, including outpatient visits, hospitalizations, and ICU stays, stratified by patient age and type of care. These costs were obtained from a previous study conducted in Colombia [23].

**Table 2:** Costs Inputs. Healthcare costs of Colombian newborns and infants with RSV infection, according to age at time of visit and type of visit in 2024

Subgroup of interest	< 1	1 - < 2	2 - < 6	6 - < 12
Hospital	$ 2,770	$ 1,768	$ 1,363	$ 1,208
ED visit	$ 36	$ 48	$ 34	$ 27
External visit	$ 171	$ 83	$ 140	$ 92

### Health outcomes and economic evaluation

We used quality-adjusted life years (QALYs) as the primary health outcome measure, with utility weights sourced from the literature and adjusted for RSV disease severity [15]. A willingness-to-pay (WTP) threshold of USD 7,491 per QALY, equivalent to Colombia’s per capita GDP, was used for the analysis [24]. We used quality-adjusted life years (QALYs) as the primary health outcome measure, with utility weights sourced from published literature and adjusted based on RSV disease severity [15]. A willingness-to-pay (WTP) threshold of USD 7,491 per QALY, equivalent to Colombia’s per capita GDP, was used for the cost-effectiveness analysis [24].

## Sensitivity analyses

We performed a one-way sensitivity analysis (OWSA) to assess the impact of key parameters, such as vaccine efficacy and RSV hospitalization rates, on model outcomes. We also conducted a probabilistic sensitivity analysis (PSA) using Monte Carlo simulations to evaluate the combined uncertainty of all model inputs.

For the probabilistic sensitivity analysis (PSA), we assigned appropriate distributions to key model parameters based on their nature and statistical properties. Beta distributions were applied to probability variables, such as vaccine efficacy, utilities and RSV incidence rates, as they are bounded between 0 and 1. Given their positive skew and non-negative values, we used gamma distributions for cost variables, including hospitalization and vaccine cost. Log-normal distributions were applied to relative risks and odds ratios to account for their multiplicative effects and to ensure positive values.

## Scenario analysis

To evaluate the impact of some model settings and assumptions on the base case results, we developed a scenario analysis. An alternative scenario with a 10-year horizon was showed, as well as variations in the discount rate in costs and benefits, using 0% and 12% for being the minimum and maximum values recommended from the national manual for cost-effectiveness analysis in Colombia [11], variations in vaccine uptake were assessed as well with ± 10% for evaluating the health impact of the uptake as cost-effectiveness is independent from this variable, and an additional scenario of an uptake of 50% to be more conservative. Additionally, another waning assumption regarding the efficacy of maternal vaccination was considered, in which efficacy wanes to 0% at 6 months.

As part of this scenario analysis, value-based prices for the intervention are presented, with a maximum of 1×GDP per capita.

## Model validation

The model underwent a validation process from both clinical and methodological perspectives. Model structure, assumptions and health states, as well as epidemiological inputs, were reviewed by an expert panel to ensure face validity and clinical relevance. Additionally, Internal validation was conducted to confirm the correct implementation of the model. The process included verification of formulas, computational accuracy and consistency between inputs and results within the statistical and software framework.

Finally, external validation was performed by comparing model outcomes with previously published cost-utility analyses and developed models. The comparison included countries like Spain [25] and Japan [26], but focusing on studies from Latin America, that were developed in similar contexts like Huerta et al. [27] from Mexico and Rey-Ares et al. [28] from Argentina. This process allowed the assessment of plausibility of the results. This comparison is developed in the discussion section.

## Results

### Base case scenario

Compared to no intervention (NI), year-round maternal RSVpreF vaccination was cost-effective at a willingness-to-pay (WTP) threshold of USD 7,491 per QALY. Implementing maternal vaccination could prevent approximately 25,781 RSV-related cases, translating into 2,315 QALYs gained in the studied cohort. The incremental cost of the intervention was around USD 5.4 million, yielding an incremental cost-effectiveness ratio (ICER) of USD $ 2,322 per QALY, which is 31% of the WTP threshold (Table 3).

The vaccination strategy demonstrated substantial reductions in RSV-related cases. As shown in Table 4, maternal RSVpreF vaccination led to a 37% reduction in RSV cases, preventing over 6,823 hospitalizations, 6,930 emergency room visits, and 12,028 outpatient visits. Additionally, the intervention could prevent 116 infant deaths. These health gains translated into 2,288 life-years (LY) and 2,315 quality-adjusted life years (QALYs).

**Table 3:** Cost-effectiveness base case results: base case results of maternal vaccination against no intervention, Colombia 2025

	RSVpreF	No intervention	RSVpreF vs. No Intervention
No. pregnant women	425,327	425,327	0
No. infants born	429,363	429,363	0
No. live births	410,223	410,223	0
Use of intervention			
No. receiving the maternal vaccine	351,868	0	351,868
% infants adequately protected*	98.6%	0	98.6%
Clinical outcomes			
No. of cases	43,597	69,378	-25,781
RSV hospitalization	7,340	14,164	-6,823
RSV ED encounter	13,407	20,337	-6,930
RSV outpatient clinic encounter	22,850	34,878	-12,028
No. of RSV-related deaths	125	241	-116
Life years (discounted)	8,083,921	8,081,633	2,288
QALYs (discounted)	7,430,788	7,428,473	2,315
Economic outcomes (millions)			
Medical care	$ 13.12	$ 25.98	-$ 12.87
Maternal vaccination	$ 18.24	$ -	$ 18.24
Total cost (Medical care + Maternal vaccination)	$ 31.36	$ 25.98	$ 5.38
ICER			
Cost per LY	Cost-effective		$ 2,349
Cost per QALY			$ 2,322

* The proportion of infants classified as “adequately protected” reflects those born to vaccinated mothers within the modeled gestational vaccination window under the assumed programmatic coverage scenario, excluding early preterm infants and those born within two weeks after vaccination

In terms of direct medical costs, they would be reduced to USD 13.12 million, which is a 49.5% reduction compared to the scenario of not vaccinating (USD 26 million). Therefore, the cost of implementing a maternal vaccination strategy with RSVpreF would amount to USD 18.2 million.

### Deterministic sensitivity analysis (DSA)

The one-way sensitivity analysis (OWSA), illustrated in Fig. 3 (Tornado Diagram), revealed that the model was most sensitive to changes in vaccine effectiveness, vaccine cost, RSV hospitalization rates, and RSV hospitalization costs. Although adjustments of ± 25% in these parameters affected the ICER, the strategy remained cost-effective across most scenarios.

**Fig. 3:**
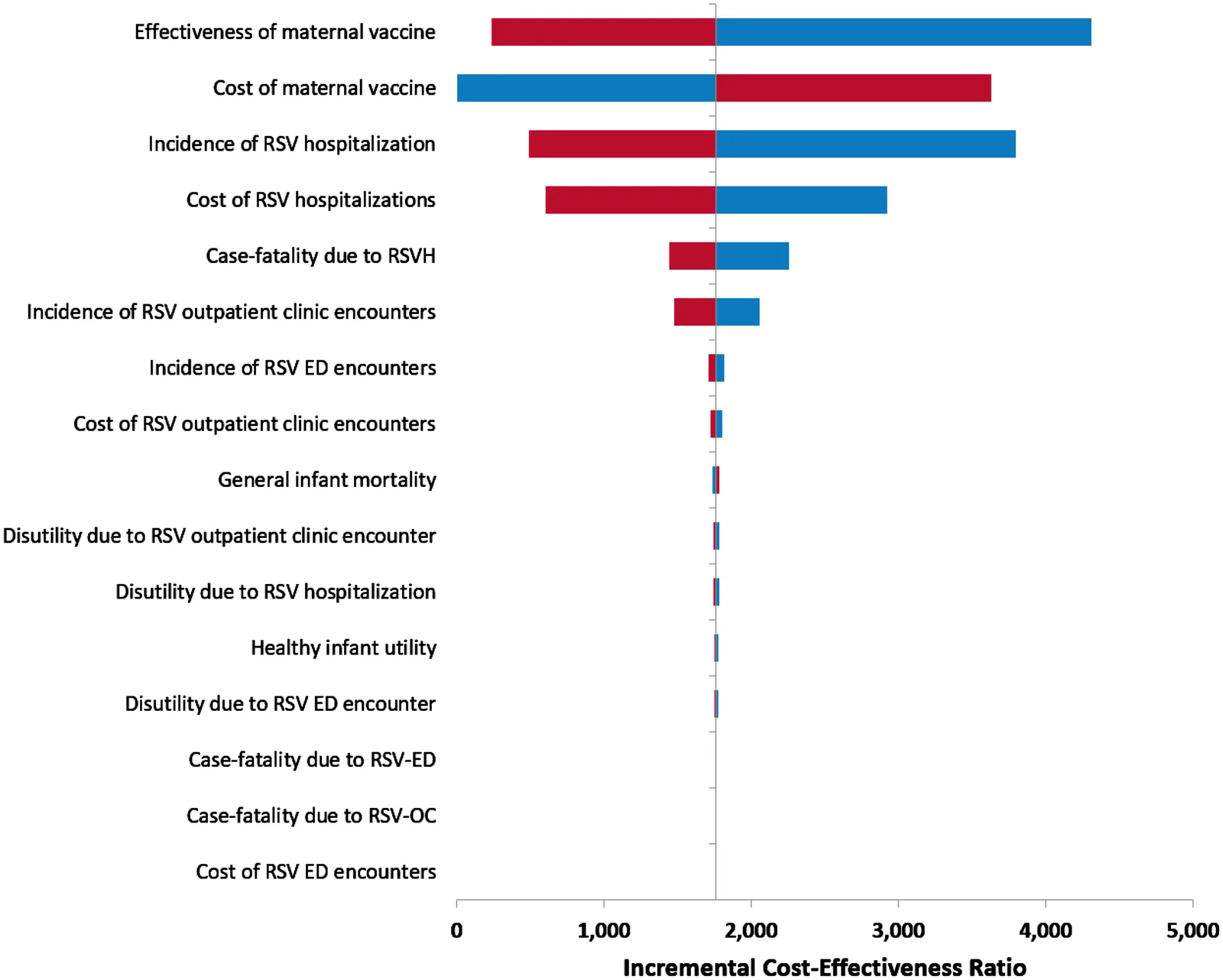
Tornado diagram: Tornado diagram for the outcomes of the DSA

### Probabilistic sensitivity analysis (PSA)

The probabilistic sensitivity analysis (PSA) assessed the combined uncertainty of model parameters. The cost-effectiveness acceptability curve (CEAC) in Fig. 4 indicates that, at the current WTP threshold, RSVpreF has an 83.5% probability of being cost-effective.

**Fig. 4:**
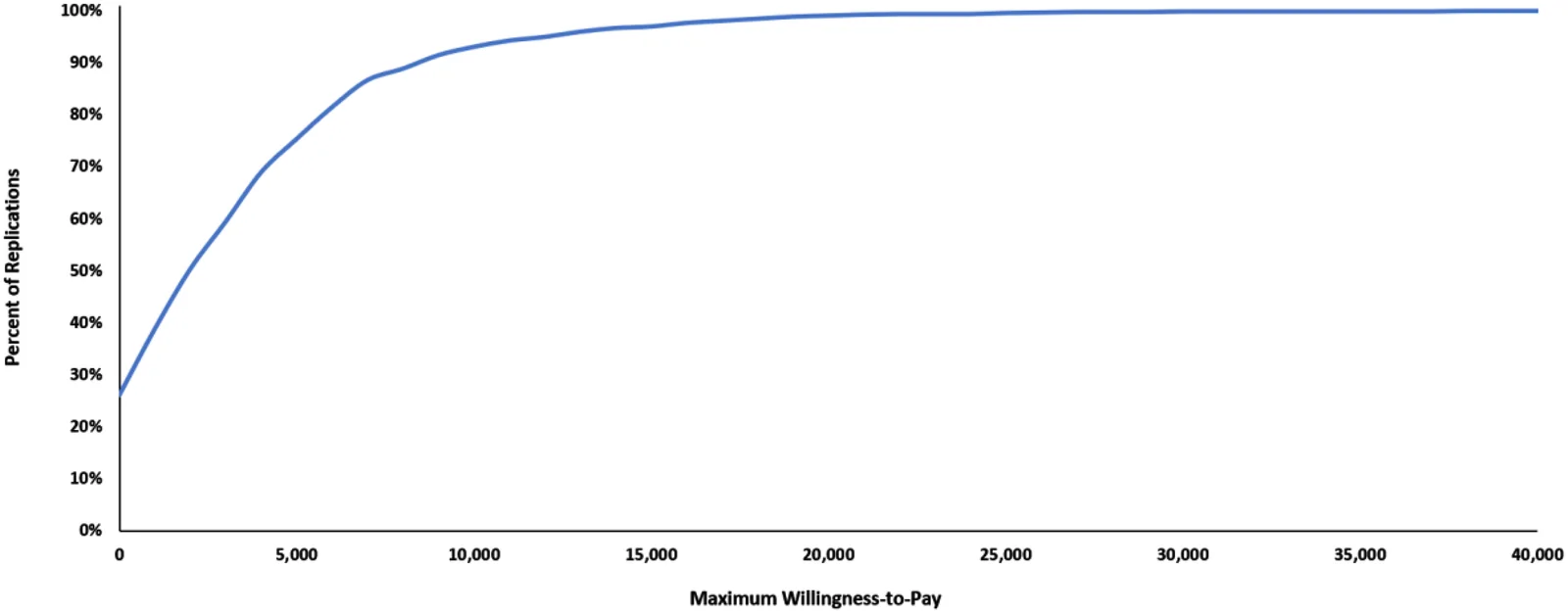
Cost-effectiveness acceptability curve: Cost-effectiveness acceptability curve for RSVpreF against no intervention

### Scenario analysis

In Table 4, the results from the scenario analysis are presented, showing that the model is robust to variations in discount rates, vaccine uptake, vaccine effectiveness waning, and time horizon, as the WTP threshold is never exceeded across all scenarios. For the discount rates, a range of 8.6% to 66.1% of 1×GDPpc is observed, obtained by setting the minimum and maximum rates recommended by national guidelines, this difference is due to the accumulation of QALYs during the life expectancy. This shows that maternal vaccinations remain cost-effective with respect to this variable. In terms of vaccine uptake, an increase or decrease of 11.5% in QALYs was observed for uptake rates of 76.7% and 96.7%, respectively. For costs, this variation is 11.4%. For the new waning assumption, the ICER increased by 10.5% points, and for the 10-year horizon, by 35.4% points; this last scenario had the highest ICER. Further details on the 10-year scenario are available in the supplementary material.

**Table 4:** Scenario analysis. Scenario analysis results for maternal vaccination compared with no intervention in Colombia, 2025

Incremental Outcomes *	Scenario 1: 0% Discount for Costs and Outcomes	Scenario 2: 12% Discount for Costs and Outcomes	Scenario 3: waning to 0% at 6 months	Scenario 4: 10-year horizon	Scenario 5: 50% uptake	Scenario 6: 76.7% uptake	Scenario 7: 96.7% uptake
Total costs discounted (Millions)	4.99	5.87	6.39	5.38	3.1	4.8	6.0
QALYs (discounted)	7,771	1,184	2,056	1,081	1,335	2,048	2,582
ICER (cost per QALY)	642	4,952	3,147	4,974	2,322	2,322	2,322
% of 1×GDPpc / QALY gained	8.6%	66.1%	41.5%	66.4%	31.0%	31.0%	31.0%

Figure 5 shows the value-based prices of interventions. This illustrates that the cost of $ 82 represents the maximum threshold at which the intervention remains cost-effective. Also, it is visible that a price below $ 34 makes the intervention dominant.

**Fig. 5:**
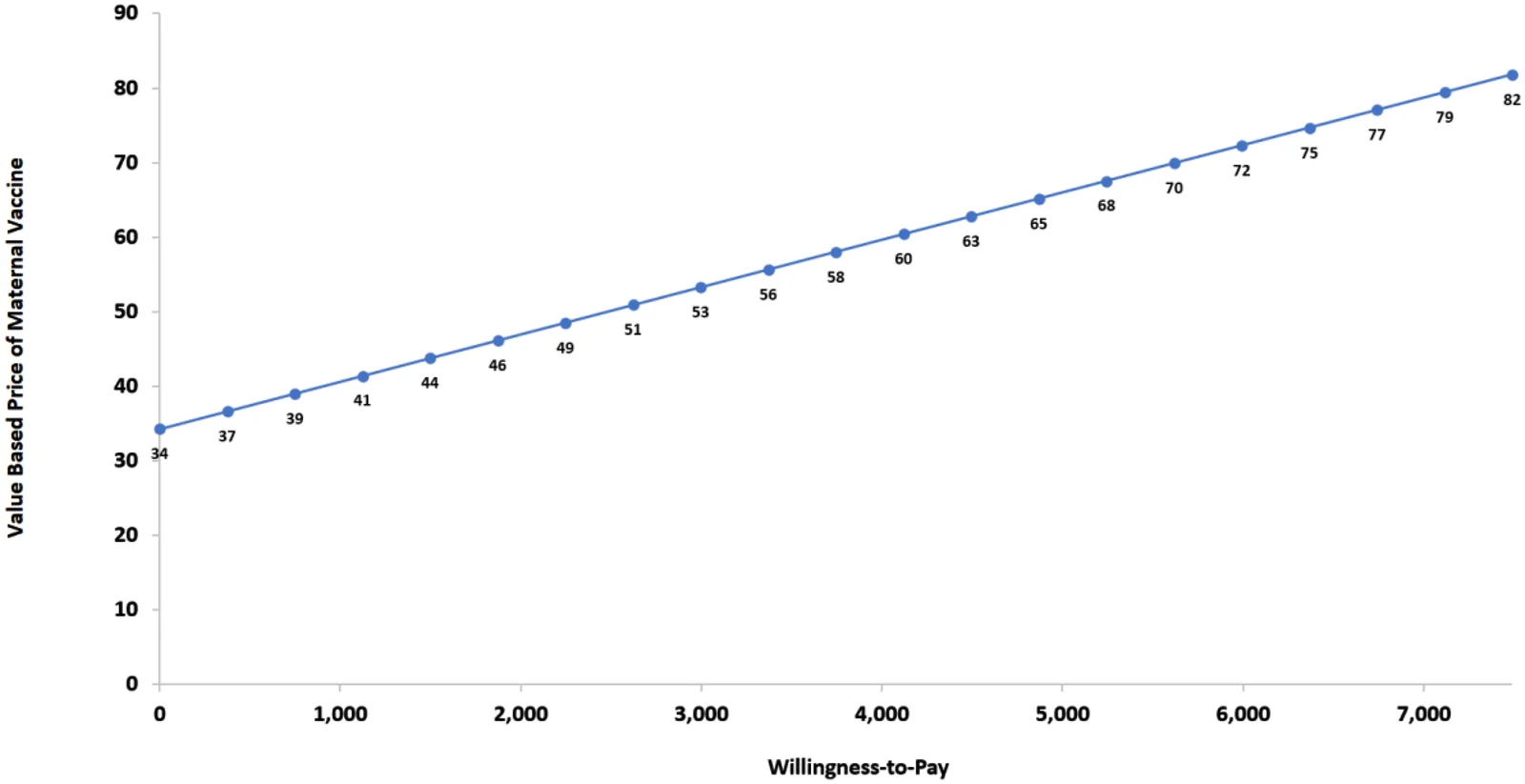
Value-Based Price of Interventions. Value-Based Price of Interventions (Maternal Vaccination vs. No Vaccination)

## Discussion

This cost-utility analysis demonstrates that maternal RSVpreF vaccination is a cost-effective intervention for reducing RSV-related morbidity and mortality in Colombian infants. With an ICER of $ 2,322 per QALY—well below the willingness-to-pay (WTP) threshold of USD 7,491—maternal vaccination represents a valuable investment in public health. These findings align with similar studies conducted in other regions, such as Argentina, Japan, Mexico, and Spain, which reported comparable cost-effectiveness ratios for maternal RSV vaccination programs [25–28].

The substantial reduction in RSV-related hospitalizations and deaths observed in this study echoes findings from Piedimonte and Perez [3], who highlighted the significant disease burden of RSV in infants and the potential of maternal vaccination to reduce severe outcomes. See [8] also emphasized the role of maternal immunization in providing passive immunity to newborns, supporting the clinical rationale behind maternal RSVpreF vaccination.

Sensitivity analysis reveals that the main drivers of ICER variation are vaccine effectiveness, vaccine price, incidence, and the cost of hospitalized RSV. However, the intervention remained cost-effective at one GDP per capita under all parameter variations. Additionally, the intervention’s cost-effectiveness was estimated at 83.5%, highlighting the robustness of the results. Finally, the value-based price analysis further supports these findings. When vaccine costs varied, the RSVpreF maternal vaccination remained cost-effective at a unit cost of up to USD 82 per dose. Rey-Ares et al. [28] and Huertas et al. [27] similarly demonstrated that maternal RSV vaccination programs remained economically viable under various vaccine cost scenarios in Argentina and México, with prices between USD 58.7 and USD 202.6 for Argentina and, USD 75.8 and USD 216 for Mexico.

Beyond cost-effectiveness, maternal RSV vaccination may have significant equity and ethical implications. The intervention protects neonates regardless of socioeconomic status, as maternal immunization is delivered through established antenatal care platforms with broad national reach. This programmatic approach aligns with National Immunization Guidelines, which emphasize reaching all demographic groups and adapting to rural and geographically dispersed populations [29], thereby helping to bridge health disparities, particularly in underserved regions where access to specialized medical care is limited. Gentile et al. [17] emphasized the disproportionate impact of RSV on low-income populations, underscoring the importance of equitable vaccination strategies.

We acknowledge that unequal access to prenatal care and variability in vaccination timing may influence real-world program performance. However, maternal immunization remains one of the most scalable strategies to confer protection from birth, particularly compared to interventions that require postnatal identification and delivery within narrower operational windows.

While this analysis evaluates maternal vaccination versus no intervention, other RSV prevention tools -particularly long-acting monoclonal antibodies administered to infants- can be considered [30]. However, they were not included due to limited comparability arising from substantial differences in clinical trials, target population and delivery platforms, an issue that has been highlighted in other studies [31]. Moreover, maternal vaccination and long-acting monoclonal antibodies could be viewed as complementary rather than competing prevention strategies, addressing different operational windows and infant risk profiles within an integrated RSV prevention framework, and the combination of both has been proven to be cost-effective [31]. A pragmatic approach in Colombia, given its high uptakes in maternal vaccination, could include monoclonal antibodies for infants not covered by maternal vaccination (e.g., missed maternal vaccination, very preterm birth, or delivery shortly after vaccination), while maternal immunization provides population-level protection starting at birth without requiring a separate newborn delivery platform.

However, this study has several limitations. Assuming static RSV incidence rates may underestimate the potential impact of epidemic fluctuations. Additionally, utility values and costs were derived from published literature and national databases, which, while robust, may not capture all nuances of local clinical practice. Another limitation regarding input parameters is that incidence estimates were extracted from regional systematic reviews instead of local data. This because RSV testing in Colombia is limited, and even when some studies has been conducted, underestimation of incidence has been noted as the principal limitation [32]. Finally, excluding indirect and societal costs underestimates the overall value of maternal vaccination with RSVpreF in Colombia, as preventing RSV cases will reduce work absenteeism among caregivers, travel costs, and out-of-pocket expenditures.

Similarly, there is uncertainty about the actual vaccination coverage rates that the country would achieve in a real-world setting, which could influence the overall public health impact of the maternal RSV vaccination program. Although high coverage rates were assumed based on existing maternal immunization programs, real-world uptake may vary due to logistical, cultural, or access-related barriers.

## Conclusions

Maternal RSVpreF vaccination offers a highly cost-effective strategy for reducing RSV-related morbidity and mortality among infants in Colombia. The health and economic benefits, combined with positive equity implications, make a compelling case for integrating this intervention into national immunization programs.

## Supplementary Information

Below is the link to the electronic supplementary material.

Supplementary Material 1

## Data Availability

Data availability is not applicable as no datasets were used or generated in the study.
