# Supplementary material for: Cost-utility of respiratory syncytial virus prefusion F (RSVpreF) maternal vaccine for disease prevention among colombian infants
Source: BMC Health Serv Res. 2026 Aug 3;26:1506. doi: 10.1186/s12913-026-15139-0 (PMC13725962; doi:10.1186/s12913-026-15139-0)
Supplement: Supplementary file 1 [file 12913_2026_15139_MOESM1_ESM.docx]

**Cost-utility of Respiratory Syncytial Virus Prefusion F (RSVpreF) Maternal Vaccine for disease prevention among Colombian infants**

**Supplementary material**

***Table S1.*** *Relative risk of developing RSV infection* *across age in months at the time of the visit, adjusted by gestational age at birth.*

| **Subgroup of interest** | **0 - < 3** | **3 - < 6** | **6 - <12** |
| --- | --- | --- | --- |
| **Full Term (> 37 wGA)** | 1,0 | 1,0 | 1,0 |
| **Late preterm (32-36 wGA)** | 1,7 | 2,5 | 1,7 |
| **Early preterm (28-31 wGA)** | 0,5 | 2,4 | 6,8 |
| **Extreme preterm (< 27 wGA)** | 0,5 | 2,4 | 6,8 |

***Table S2.*** *Relative risk of infant mortality due to RSV infection across age in months at the time of the visit, adjusted by gestational age at birth.*

| **Subgroup of interest** | **< 1** | **1 - < 6** | **6 - <12** |
| --- | --- | --- | --- |
| **Full Term (> 37 wGA)** | 1,0 | 1,0 | 1,0 |
| **Late preterm (32-36 wGA)** | 13,3 | 4,9 | 2,3 |
| **Early preterm (28-31 wGA)** | 13,3 | 4,9 | 2,3 |
| **Extreme preterm (< 27 wGA)** | 273,4 | 37,2 | 49,0 |

**Added Scenario: 10-year horizon**

**In this section, a scenario of a 10-year horizon is presented for RSVpreF vaccination against no intervention.**

**Results**

With a 10-year horizon, compared to no intervention (NI), year-round maternal RSVpreF vaccination was cost-effective at a willingness-to-pay (WTP) threshold of USD 7,491 per QALY. Implementing maternal vaccination could prevent approximately 25,781 RSV-related cases, translating into 1,081 QALYs gained in the studied cohort. The incremental cost of the intervention was around USD 4 million, leading to an incremental cost-effectiveness ratio (ICER) of $ 4,974 per QALY, which is less than half the WTP threshold (66.4%).

**Health Outcomes:** The vaccination strategy demonstrated substantial reductions in RSV-related cases. As shown in *Table S3*, maternal RSVpreF vaccination led to a 37% reduction in RSV cases, preventing over 6,823 hospitalizations, 6,930 emergency room visits, and 12,028 outpatient visits. Additionally, the intervention could prevent 116 infant deaths. These health gains translated into 892 life-years (LY) and 1,081 quality-adjusted life years (QALYs).

***Table S3****. Deterministic results of maternal vaccination against no intervention, Colombia 2025.*

|  | RSVpreF | No intervention | RSVpreF vs. No Intervention |
| --- | --- | --- | --- |
| No. pregnant women | 425,327 | 425,327 | 0 |
| No. infants born | 429,363 | 429,363 | 0 |
| No. live births | 410,223 | 410,223 | 0 |
| Use of intervention | | | |
| No. receiving the maternal vaccine | 351,868 | 0 | 351,868 |
| % infants adequately protected | 98.6 % | 0 | 98.6 % |
| Clinical outcomes | | | |
| No. of cases | 43,597 | 69,378 | -25,781 |
| RSV hospitalization | 7,340 | 14,164 | -6,823 |
| RSV ED encounter | 13,407 | 20,337 | -6,930 |
| RSV outpatient clinic encounter | 22,850 | 34,878 | -12,028 |
| No. of RSV-related deaths | 125 | 241 | -116 |
| Life years (discounted) | 3,216,099 | 3,215,208 | 892 |
| QALYs (discounted) | 3,128,326 | 3,127,245 | 1,081 |
| Economic outcomes (millions) | | | |
| Medical care | $ 13.12 | $ 25.98 | -$ 12.87 |
| Maternal vaccination | $ 18.24 | $ - | $ 18.24 |
| Total cost (Medical care + Maternal vaccination) | $ 31.36 | $ 25.98 | $ 8.94 |
| ICER |  |  |  |
| Cost per LY | Cost-effective |  | $ 6,029 |
| Cost per QALY |  |  | $ 4,974 |

**Economic Outcomes:** In terms of direct medical costs, they would be reduced to USD 13.1 million, which is a 49.5% reduction compared to the scenario of not vaccinating (USD 26.0 million). Additionally, the cost of implementing a maternal vaccination strategy with RSVpreF would amount to USD 18.2 million.

**Deterministic Sensitivity Analysis (DSA):** The one-way sensitivity analysis (OWSA), illustrated in *Figure S1* (Tornado Diagram), revealed that the model was most sensitive to changes in vaccine effectiveness, RSV hospitalization rates, and RSV hospitalization costs. Although adjustments of ±25 % in these parameters affected the ICER, the strategy remained cost-effective across most scenarios.

***Figure S1. Tornado diagram****. Tornado diagram for the outcomes of the DSA.*

**Probabilistic Sensitivity Analysis (PSA):** The probabilistic sensitivity analysis (PSA) assessed the combined uncertainty of model parameters. The cost-effectiveness acceptability curve (CEAC), shown in *Figure S2*, indicated that at the current WTP threshold, RSVpreF has a 61.1 % probability of being a cost-effective intervention.

***Figure S2. Cost-effectiveness acceptability curve****. Cost-effectiveness acceptability curve for RSVpreF against no intervention.*
